# THE IMPACT OF INFORMAL CARE ON FORMAL CARE UTILIZATION AMONG ELDERLY AMERICANS

**DOI:** 10.1093/geroni/igad104.3735

**Published:** 2023-12-21

**Authors:** Yemiao Ke, Yun Zhang

**Affiliations:** Stony Brook University, Stony Brook, New York, United States; Columbia University, New York City, New York, United States

## Abstract

**Objectives:**

This research aims (1) to examines the impact of informal care on the utilization of formal care among elderly Americans, and (2) to investigate how probable dementia affect this relationship.

**Method:**

Using cross-sectional data from the National Health and Aging Trends Study (NHATS 2011 & 2015), we build a correlated Tobit model to analyze the quantity of informal and formal care received by elderly individuals, which not only handles the censored nature of the dependent variables, but also allows a more flexible error structure. To address endogeneity concerns, instrumental variables derived from the characteristics of the elderly’s children are incorporated.

**Result:**

Participants who did not live in long-term care facilities (N = 7,090) were included. Our estimation results indicate that when monthly hours of informal care received are below 83 hours, the monthly hours of formal care decrease with an increase in informal care. Conversely, when monthly hours of informal care received exceed 83 hours, the monthly hours of formal care increase with additional informal care. Probable dementia increases the monthly hours of informal care by 31.2 hours and the monthly hours of formal care by 6.4 hours.

**Conclusion:**

Informal care substitutes for formal care when elderly individuals require only a low to moderate level of informal care. However, informal and formal care become complementary when the elderly requires more intensive care. Probable dementia has a significant impact on both informal care and formal care.

